# Effect of behavioral change intervention around new-born care practices among most marginalized women in self-help groups in rural India: analyses of three cross-sectional surveys between 2013 and 2016

**DOI:** 10.1038/s41372-019-0358-1

**Published:** 2019-04-02

**Authors:** Niranjan Saggurti, Akash Porwal, Yamini Atmavilas, Monika Walia, Rajshree Das, Laili Irani

**Affiliations:** 1 Population Council, New Delhi, India; 2 Bill and Melinda Gates Foundation, New Delhi, India; 3 Project Concern International, New Delhi, India

**Keywords:** Developing world, Population screening

## Abstract

**Objective:**

To assess the effects of new-born care intervention through self-help groups in improving new-born healthcare practices in rural India.

**Methods:**

A quasi-experimental design was used to evaluate behavioral change intervention integrated in >25,000 microfinance-based self-help groups in rural Bihar. Three rounds of cross-sectional surveys were conducted to understand the impact of intervention on new-born healthcare practices by talking to women who delivered a baby in the last 6 months.

**Results:**

Intervention groups showed greater improvement than control groups in the timely initiation of breastfeeding (adjusted odds ratio (AOR) = 6.3, 95% CI: 2.8, 14.3), exclusive breastfeeding on day 1 (AOR = 4.3, 95% CI: 1.9, 9.9), initiation of skin-to-skin care (AOR = 1.9, CI: 1.0, 3.8), and delayed bathing (AOR = 2.8, 95% CI: 1.4, 5.9) with greater effect of on home deliveries where clinical care is often absent.

**Conclusion:**

Sharing messages on appropriate new-born practices through self-help groups improve new-born care practices.

## Background

Globally, an estimated three million children die in the first 28 days of life [1]; more than one in every five global deaths happens in India [2]. Most neonatal deaths occur at home in low resource settings [3] against a backdrop of poverty [4, 5] and harmful new-born care practices [6]. India continues to have high neonatal mortality with considerable variations across states [7]. More than 50% of neonatal deaths happen on the first day of life [8]; studies have recommended that such deaths can be prevented with an initial focus on preventive family and community interventions and optimal care [4].

The establishment of women’s groups, a community participation approach, has proven to be a promising community-based platform in improving new-born care practices in the community [9, 10] and reducing new-born mortality [3, 11] in India and elsewhere [12]. Women’s groups bring women with similar needs together to discuss topics that are of concern to them, helping them devise their own solutions. Many interventions implemented through such groups are based on a participatory learning and action cycle approach, in which group members identify and prioritize maternal and new-born health problems in the community, collectively select strategies to address these problems, implement the strategies, and assess the results. The nature of such groups, for example, microfinance-related activities among self-help groups (SHGs), creates solidarity and social capital along with financial capital, since members meet regularly for transactions, trainings, and conversations [13]. Macro and synthesis analyses of data from the national health survey from 601 districts in India against the data on volume of SHGs in India indicates that women from villages with SHGs were 19% (odds ratio (OR): 1.19, confidence interval (CI): 1.13–1.24) more likely to have delivered in an institution, 8% (OR: 1.08, CI: 1.05–1.14) more likely to have fed colostrum to their new-borns, and have knowledge of (OR: 1.48, CI 1.39–1.57) and utilized (OR: 1.19, CI 1.11–1.27) family planning products and services [14]; however, the study results are only indicative as there is no evidence to suggest that health information was disseminated through the SHGs. A cluster-randomized controlled trial conducted in India, the “Ekjut trial”, showed a strong effect of women’s groups on reduction of the neonatal mortality rate (NMR) in the population in whom the trial was conducted (a 45% decline in years 2 and 3 of the intervention) [15]. Hence, more evidence is needed on the role of SHGs to affect maternal and child health outcomes.

Bihar is one of the poor states in northern India with a population of 100 million people [7]. Although, the state’s NMRs are below the national average [16], most of the neonatal deaths happen within the first 3 days of life [17]. Given that most of the neonatal deaths are happening among women belonging to poor socioeconomic conditions [18], it calls for a focused intervention. Coincidentally, in 2012, Bihar state government had committed to create and nurture women SHGs throughout the state [19], particularly in rural and remote areas. Given the evidence from small-scale program interventions in other parts of India and countries in south Asia around implementation of health intervention through SHGs, the need to improve new-born health outcomes through scaled intervention grew attention in the state. Unfortunately, no model in India has ever tested the improvement in new-born healthcare practices through SHGs at scale and at different time points; the only rigorous evaluation of the intervention that engaged SHGs was a secondary data analysis but it did not assess the effects of group membership over time and also not at the scale [14]. The current study evaluates a health intervention in which a dedicated two module program on new-born healthcare practices was delivered by community workers to women in SHGs to improve the existing new-born health practices in rural areas.

## Methods

### Program description

In 2011, the Bill and Melinda Gates Foundation implemented a set of innovations under the Ananya program in eight districts of Bihar in order to reduce the maternal and neonatal mortality through a partnership with the Government of Bihar [20]. The Ananya program’s design combined both supply- and demand-side interventions to improve reproductive, maternal, newborn and child health (RMNCH) services and outcomes. The supply-side intervention included a focus on strengthening community outreach and facility services. Community outreach workers, also called frontline workers (FLWs include three categories of workers—accredited social health activists [ASHAs], anganwadi workers and auxiliary nurse midwives) operating in rural Bihar were trained to implement critical maternal and new-born interventions. They were also given job-aid kits, which reinforced their training in interpersonal communication, and messages on positive behaviors in order to support discussions with eligible women in their local geographies. The other element of the supply-side intervention included improvement of basic emergency obstetric care services at facility level. The supply-side intervention was implemented uniformly in rural areas across the study districts, covering almost all facilities and FLWs.

The major element of the demand-side intervention in Ananya entailed using an innovative program with SHGs called Parivartan. This program entailed forming and nurturing a total of 19,000 health-focused SHGs each with a membership of 10–12 women of reproductive age belonging to the most marginalized communities, i.e., scheduled castes, scheduled tribes, and pasmanda Muslims (considered to be a socially backward Muslim community). At any given time point, each SHG had about 1–2 pregnant women, and an additional 1–2 women with children below 2 years of age. Health “integration” within intervention SHGs included eight weekly cycles of participatory behavioral communications using different thematic modules, the details of which are reported elsewhere [21]. The intervention session was delivered by a trained community worker (woman, non-FLW). She was often a young lady from the local rural community with basic education and was interested in imparting health knowledge to the women in her community. The community workers were not an official member of any SHG but attended the group meetings whenever a health module was to be delivered. They were not expected to share information through other avenues and no other educational efforts were underway in the study areas. FLWs attended some health discussions within intervention SHGs in few of the geographies.

### Intervention condition

The intervention that is of relevance to this article included two modules on new-born health practices and was delivered across 1–2 months in all the groups. Module 1 focused on immediate postnatal behaviors after delivery like the need for delayed bathing of child, skin-to-skin care, clean cord care, and timely initiation of breastfeeding. Health messages relevant to mentioned key behaviors were delivered using a story play narrating the importance and right way of practicing neonatal care behaviors. Along with the story play, flip cards were used to re-emphasize the importance of practicing neonatal care behaviors. Each flip card consisted of a behavior along with a key message. Module 2 focused on exclusive breastfeeding till 6 months of child’s age. The module covers the importance of behavior through a cardboard puzzle game, which was intended to generate awareness on the ways of keeping the child healthy. In addition, flip cards were used with key messages around exclusive breastfeeding. Each module was delivered in one out of four weekly group meetings and was repeated in the remaining three meetings of the month. Each module was implemented once over 2–3 months in year 1 and again in year 2 of the project. There was no repeat of messages in year 3. The theory of change is that receiving the information in an SHG meeting would facilitate a conversation within a home environment with an eligible woman, mother-in-law, husband, etc. around the practice of a new healthy behavior. It would further muster the support of fellow SHG members in helping the pregnant woman and mother of a neonate plan for how she could take up the practice of the new behavior further facilitating uptake.

### Control condition

Groups in the control condition were part of government nurtured SHGs (approximately 6000 SHGs) that existed at the start of the Parivartan program in 2012. The government nurtured SHGs provided financial literacy, and savings support and services to the members of SHGs. The control condition did not have any structured focus on new-born health behaviors.

### Evaluation methodology

This evaluation study uses three rounds of cross-sectional surveys conducted in 2013, 2014, and 2016 among women from SHGs and belonging to scheduled castes/tribes and pasmanda muslims (considered to be lowest social class in the society) in eight districts of rural Bihar. The surveys were conducted as part of a large-scale program implemented in Bihar by Project Concern International (PCI). The information obtained in the repeated cross-sectional surveys is used to both track the progress of the program, and make mid-course corrections, as needed. The surveys have monitored a range of reproductive, maternal, new-born, child health issues, including the extent to which the SHG mobilization and accountability had an impact.

The first round of survey was conducted from April to June 2013 in 35 blocks (out of the total 67 intervention blocks at the time of survey) of eight Bihar districts, namely Patna, Saharsa, East Champaran, West Champaran, Samastipur, Begusarai, Gopalganj, and Khagaria. The sample size was estimated to provide robust estimates for prevailing levels of three key family health and sanitation indicators: awareness of sanitation, safe delivery, and at least three antenatal care (ANC) visits. The sample size was calculated to provide estimates with 95% confidence and 5% margin of error. A two-stage cluster sampling design selected study participants from all eight districts. At the first stage, 35 of 67 blocks (the initial project areas) were randomly selected with the use of random numbers generated using MS-Excel. Of the 35 selected blocks, in 27 blocks, Parivartan program was implemented. In the remaining eight blocks, the government worked with SHGs on small savings and credit, and those groups were selected as controls. In the second stage, the SHGs were systematically selected within each selected block. The SHG lists that were available with the program were used as a sampling frame for the selection of SHGs. The first SHG was selected using a random number followed by every *n*th SHG chosen from the list.

The remaining two survey rounds followed the same sampling design to identify the groups and the women from the groups. A subset of groups included in the first round of survey was included in 2nd and 3rd round (common groups are about 345) of survey to study the changes longitudinally. Although, the groups were common, the women were different as the sample included in the survey are the new mothers with a child less than a year old. The analyses in this study use data from all groups in each round of the survey and treat it as a cross-sectional sample at various time points.

Within selected groups, trained female research staff approached all women to determine whether a married woman aged 18–49 years with a child <1-year old (used as a first level of screening) was available. If a woman with the specified criteria was available, a private space was identified within the house or nearby place for the consent process. A comprehensive informed consent process was followed, with respondents informed about the study, including the interview’s duration (approximately 45 min), and their queries addressed before written consent was taken. In cases where respondents were illiterate or did not want to sign the consent form, verbal consent was taken. A copy of their written consent was provided to respondents for their record. Research staff clarified the study procedures and asked the respondent whether she would like to participate in the broader evaluation, which involved a detailed interview to assess the intervention’s reach and effectiveness. Participants were not given any monetary compensation for their time but were provided information on incentives provided by health programs and financial services available in their area. Interviews were conducted in Hindi, a local language of the participants. All study procedures were reviewed and approved by the Institutional Review Board of the Population Council.

**Fig. 1:**
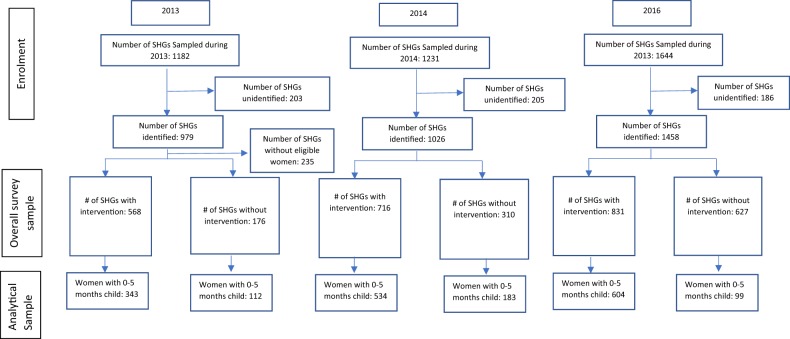
CONSORT chart on sample recruitment

After acquiring written/oral informed consent, the 40-min interview included a variety of questions on ANC, institutional delivery, new-born health practices, contraception, child morbidity and treatment seeking behavior, community collectivization, self-efficacy, agency and exposure to the program (this issue was examined particularly in the 2nd and 3rd round of the surveys). Over the three rounds of survey, the number of eligible women interviewed were: 2407 (in 2013), 2237 (in 2014), and 2974 (in 2016) (Fig. 1). Overall, during all three rounds, none of the sampled cases refused to participate in the survey. However, non-response of 14% (2013), 17% (2014), and 11 % (2016) was reported due to non-availability of eligible women at home.

### Measures

Survey measures included single items on socioeconomic and demographic characteristics (e.g., age, education, social status, engagement in economic activity, and number of children ever born). The outcome measures used for assessing new-born healthcare practices were single questions in the survey instrument, except for the clean cord care practices. The clean cord care measure was calculated by combining the scores based on the following questions: (a) whether new instrument was used to cut the umbilical cord; (b) whether new thread was used to tie the cord; (c) whether anything was applied on the cord; and (d) whether anything was applied on the stump after the cord dropped off. Clean cord care score was calculated and coded as “Yes”, if all the above-mentioned behaviors were rightly practiced. Right behaviors considered for clean cord care measure were: cutting of cord with new blade and tying of cord with new thread and nothing at all applied on cord (including chlorhexidine) and nothing applied on stump after cord is dropped off (including chlorhexidine). Score was coded as “No” if any one of the above behaviors was not practiced correctly.

The questions asked in the survey instrument for the remaining measures are: (1) initiation of skin-to-skin care—whether child was placed on the chest of mother immediately after delivery for at least an hour; (2) timely initiation of breastfeeding—duration after which child was given breastmilk for the first time after birth (within 30 min of birth); (3) exclusive breastfeeding on day 1—whether child was given anything else other than breastmilk on the 1st day of birth; (4) delayed bathing of child—whether child was bathed after 72 h of birth (yes, no). The primary independent variable in the analyses was SHGs with new-born health messaging intervention (intervention groups) versus the SHGs without such a program (control groups).

### Statistical analyses

Outcome analyses used an intention-to-treat approach and assessed the effects of new-born health intervention within SHGs on practices. Chi-squared analyses were conducted to determine the demographic differences between the participants recruited for survey at different time points. Logistic regression models were constructed with time, type of intervention, and time × type of intervention. STATA version 13.0 (StataCorp, College Station, TX, USA) was used to conduct all statistical analyses.

## Results

Mean age of participants ranged between 24.4 and 25.9 years in the intervention and control groups, and the mean age of the overall sample was 26 (±4.7) years (Table 1). Over time, with the change in survey rounds (from 2013 to 2016), the percent with formal education has increased across both intervention (from 18 to 20%, *p* < 0.001) and control (from 13 to 32%, *p* = 0.003) groups. With the survey round, the duration of association with SHGs has increased significantly in both intervention (*p* < 0.001) and control (*p* < 0.001) groups.

**Table 1:** Sociodemographic characteristics of participants over time

	Intervention groups				Control groups				*p*-Value between intervention vs control groups in 2013^*^
	2013	2014	2016	*p*-Value^*^	2013	2014	2016	*p*-Value^*^	
Sample size (# of women)^a^	343	534	604	NA	112	183	99	NA	NA
Average age in years (±SD)	24.4 (±4.3)	25.9 (±4.8)	25.9 (±4.4)	<0.001	25.7 (±5.1)	26.4 (±4.8)	26.6 (±4.4)	0.349	0.495
Percent with formal education	18.1	16.3	20.4	0.206	12.5	19.3	31.3	0.003	0.515
Percent SC/ST households	86.1	81.1	80.9	0.107	77.7	88.5	90.9	0.009	0.054
Percent engaged in economic activity	36.0	34.8	35.3	0.953	52.0	36.6	28.3	0.002	0.158
Average number of children (±SD)	2.9 (±1.7)	3.2 (±1.8)	3.4 (±1.6)	<0.001	3.4 (±1.8)	3.4 (±1.6)	3.3 (±1.5)	<0.001	0.145
Average duration of association with SHGs in months (±SD)	4.8 (±1.9)	15.6 (±4.9)	33.1 (±13.1)	<0.001	18.7 (±11.4)	17.8 (±13)	29.5 (±9.1)	<0.001	0.057

*Test for difference between means, proportion

^a^Women who have <6 months child

The proportion of women reporting practices around essential new-born care has increased significantly over time in case of intervention groups (Table 2). Among the control groups, there was a significant improvement in case of clean cord care, initiation of skin-to-skin care, and delayed bathing. The increase in new-born care practices within intervention groups was significant even if the woman delivered a baby at home. For instance, the initiation of skin-to-skin care has increased from 21% in 2013 to 45% in 2016 in the intervention groups (*p* < 0.001). Similarly, the timely initiation of breastfeeding has increased from 46% in 2013 to 87% in 2016 (*p* < 0.001).

**Table 2:** Trends in neonatal care practices among women in intervention and control groups in Bihar, 2013–2016

Indicators	Intervention group (*N* = 1481)				Control group (*N* = 394)			
	2013	2014	2016	*p*-Value^*^	2013	2014	2016	*p*-Value^*^
Overall								
Clean cord care (%)	19.8	76.2	72.2	**<** **0.001**	16.1	63.4	54.5	**<** **0.001**
Initiation of skin-to-skin care (%)	33.5	62.0	60.4	**<** **0.001**	29.4	46.4	41.4	**0.015**
Timely initiation of breastfeeding (%)	64.4	84.3	89.6	**<** **0.001**	83.9	76.2	76.3	0.266
Exclusive breastfeeding on day 1 (%)	70.8	89.0	91.7	**<** **0.001**	81.2	74.0	80.8	0.224
Delayed bathing (%)	18.4	53.2	61.8	**<** **0.001**	18.7	30.6	44.4	**<** **0.001**
Place of delivery: health facility								
Clean cord care (%)	22.2	80.5	75.3	**<** **0.001**	14.3	71.3	61.3	**<** **0.001**
Initiation of skin-to-skin care (%)	39.3	75.3	67.2	**<** **0.001**	40.0	60.8	45.3	**0.007**
Timely initiation of breastfeeding (%)	73.8	87.1	90.9	**<** **0.001**	89.6	87.8	81.2	0.285
Exclusive breastfeeding on day 1 (%)	78.6	91.0	91.2	**<** **0.001**	85.7	88.7	84.0	0.632
Delayed bathing (%)	20.9	57.1	66.8	**<** **0.001**	22.1	36.5	44.0	**0.015**
Place of delivery: home								
Clean cord care (%)	14.6	67.1	65.0	**<** **0.001**	20.0	50.0	33.3	**0.010**
Initiation of skin-to-skin care (%)	21.1	33.5	44.8	**<** **0.001**	8.6	22.1	29.2	0.114
Timely initiation of breastfeeding (%)	45.9	78.2	86.6	**<** **0.001**	71.4	57.3	62.5	0.378
Exclusive breastfeeding on day 1 (%)	54.1	84.7	92.9	**<** **0.001**	71.4	48.5	70.8	**0.035**
Delayed bathing (%)	12.8	44.7	50.3	**<** **0.001**	11.4	20.6	45.8	0.007

^*^Chi-square test

The bold values indicate the results that had statistical significance

Multiple logistic regression analyses indicated that the increase over time in new-born care practices among women from intervention groups are much better when compared with women from control groups, even after controlling for sociodemographic and economic characteristics. For instance, the likelihood of change from 2013 to 2014 among women in the intervention groups compared with control groups was significant in timely initiation of breastfeeding (adjusted odds ratio (AOR) = 4.2, 95% CI: 2.1, 8.6), exclusive breastfeeding on day 1 (AOR = 4.7, 95% CI: 2.3, 9.5), and delayed bathing (AOR = 3.1, 95% CI: 1.6, 6.1) (Table 3). Similar changes were observed from 2013 to 2016 among women in intervention groups as compared with their counterparts in control groups.

**Table 3:** Impact of health integration intervention (AOR, 95% CI) in SHGs on neonatal health practices in Bihar, India

Characteristics^a^	Clean cord care	Initiation of skin-to-skin care	Timely initiation of breastfeeding	Exclusive breastfeeding on day 1	Delayed bathing
By time					
2013 (ref.)					
2014	**12.1 (8.9–16.4)**	**2.9 (2.2–3.7)**	**1.8 (1.3–2.4)**	**1.9 (1.4–2.6)**	**4.4 (3.3–5.9)**
2016	**11.4 (7.8–16.7)**	**2.8 (2.0–4.0)**	**2.0 (1.3–3.0)**	**2.5 (1.6–4.0)**	**8.7 (6.0–12.6)**
By type					
Control groups (ref.)					
Intervention groups	**1.9 (1.5–2.3)**	**1.8 (1.5–2.3)**	**1.3 (1.0–1.7)**	**1.9 (1.4–2.6)**	**2.3 (1.8–2.9)**
By type × time					
Control group in 2013 (ref.)					
Intervention group in 2014	1.4 (0.7–2.7)	1.6 (0.9–2.9)	**4.2 (2.1–8.6)**	**4.7 (2.3–9.5)**	**3.1 (1.6–6.1)**
Intervention group in 2016	1.8 (0.7–2.2)	**1.9 (1.0–3.8)**	**6.3 (2.8–14.3)**	**4.3 (1.9–9.9)**	**2.8 (1.4–5.9)**

*AOR* adjusted odds ratio, *CI* confidence interval

Adjusted for age, education, caste/religion, occupation, number of children ever born, length of group association

^a^Time, type, type × time are three separate models in multivariate analyses

The bold values indicate the results that had statistical significance

Further, the analyses by place of delivery present differential effects in new-born care practices. For example, for women who delivered at a health facility, the health integration intervention had a positive impact on initiation of skin-to-skin care, and timely initiation of breastfeeding only in 2016 but not in 2014 (Table 4). However, for women who delivered a baby at home, the intervention had significant impact on timely initiation of breastfeeding and exclusive breastfeeding on first day in both 2014 and 2016.

**Table 4:** Impact of health integration intervention (AOR, 95% CI) in SHGs on neonatal health practices by place of delivery, India

Characteristics^a^	Clean cord care	Initiation of skin-to-skin care	Timely initiation of breastfeeding	Exclusive breastfeeding on day 1	Delayed bathing
Place of delivery: health facility					
By time					
2013 (ref.)					
2014	**15.9 (10.9–23.2)**	**3.7 (2.7–5.1)**	**1.6 (1.0–2.4)**	**1.9 (1.2–3.1)**	**4.2 (2.9–5.8)**
2016	**15.2 (9.4–24.5)**	**2.3 (1.5–3.5)**	1.3 (0.7–2.2)	1.5 (0.8–2.7)	**6.9 (4.5–10.6)**
By type					
Control groups (ref.)					
Intervention groups	**1.8 (1.3–2.4)**	**1.8 (1.4–2.4)**	0.9 (0.6–1.4)	1.3 (0.9–1.9)	**2.3 (1.7–3.0)**
By type × time					
Control group in 2013 (ref.)					
Intervention group in 2014	0.9 (0.8–1.6)	1.8 (0.9–3.7)	2.5 (0.9–7.1)	1.8 (0.6–5.0)	**2.7 (1.3–6.0)**
Intervention group in 2016	1.3 (0.5–3.3)	**2.4 (1.1–5.2)**	**5.9 (1.9–17.9)**	3.1 (1.1–9.1)	**3.2 (1.4–7.4)**
Place of delivery: home					
By time					
2013 (ref.)					
2014	**8.2 (4.8–14.1)**	**2.2 (1.3–3.8)**	**2.5 (1.5–4.0)**	**2.1 (1.3–3.5)**	**5.5 (3.1–9.9)**
2016	**6.3 (3.2–12.4)**	**4.3 (2.2–8.5)**	**3.8 (1.9–7.5)**	**6.1 (2.8–13.4)**	**14.5 (6.9–30.6)**
By type					
Control groups (ref.)					
Intervention groups	**2.1 (1.3–3.1)**	**2.3 (1.4–3.8)**	**2.0 (1.3–3.0)**	**3.5 (2.3–5.6)**	**2.3 (1.4–3.6)**
By type × time					
Control group in 2013 (ref.)					
Intervention group in 2014	2.3 (0.7–7.5)	0.6 (0.1–2.5)	**6.1 (2.1–17.8)**	**11.6 (3.9–34.4)**	3.2 (0.8–12.8)
Intervention group in 2016	3.8 (0.9–15.3)	0.8 (0.2–4.0)	**10.2 (2.7–38.2)**	**11.1 (2.7–45.6)**	1.7 (0.4–7.5)

*AOR* adjusted odds ratio, *CI* confidence interval

Adjusted for age, education, caste/religion, occupation, number of children ever born, length of group association

^a^Time, type, type × time are three separate models in multivariate analyses

The bold values indicate the results that had statistical significance

## Discussion

The findings indicate that the behavioral change program implemented through SHGs has shown a substantial improvement in essential new-born care practices among most marginalized population in rural India. Improvement in select indicators of new-born care is evident even within 1 year of intervention. And those behaviors sustained to have an impact even after 3 years indicating the utility of simple behavioral change programs introduced through structured women’s collectives. These results are consistent with existing evidence from eastern India on new-born care practices [15, 22]. However, most of the existing evidence was from controlled small-scale settings. The current study adds to this evidence in highlighting the possibility of achieving good new-born care practices in scaled-up interventions, and in areas with high levels of poverty and lack of enough health infrastructure [23] through improvements in knowledge [24] and cohesion between members of the groups [21, 25].

Differential outcomes for participants who were part of the intervention groups depending on their place of delivery is encouraging. While, one would expect to see positive essential new-born care for all deliveries happening in institutions, results of this study had shown that the timely initiation of breastfeeding and exclusive breastfeeding practices are likely to improve significantly even if deliveries are taking place at home. Such a change in behavior gains importance given the importance of timely initiation of breastfeeding on saving lives of neonates [26].

These findings provide further evidence to the call for promoting community-focused interventions to bring about early success in reducing new-born mortality while working to strengthen health systems [4]. The strategy used by the program to integrate new-born care messaging within SHGs complements the interventions within the health systems being implemented at the state and national levels [27]. Evidence around the role of community participation and/or SHGs on maternal and new-born healthcare is evolving [10, 28]. Studies have shown that community participation particularly through SHG mechanisms show an avenue to voice their concerns and provide a unique space in which solidarity is created through promoting shared visions and goals and combining collective strengths [14]. Post-hoc analyses of what may have led to the change in the new-born care practices among women from intervention groups has indicated the improved collective efficacy (sense of being together) wherein discussion about new-born care practices took place between members of the group.

The process of structured health messaging around new-born care practice and encouraging women to further discuss it among themselves seems to have worked well with illiterate and marginalized population groups in the program geographies. Prior to the receipt of health messages through the modules, the women in groups went through different phases of togetherness with the program facilitation. Not only that women living in the same village came together to become part of the homogenous (on the basis of caste or economic criteria) group, but also heard from the program team repeatedly about the need for better reproductive, maternal, new-born and child care practices in order to save mother and new-born lives. In addition, women’s sharing of correct practices during health discussions about new-born care behaviors particularly around breastfeeding, delayed bathing of the baby, and clean cord care helped improve not only improve the knowledge of women but improved bonding between members of the group. Collective efficacy playing an important role in changing harmful practices were demonstrated in maternal and child health programs like these [21, 24, 25], as well as in HIV prevention programs [29–33].

The evidence from this program evaluation is important given the paucity of evidence around the impact of scaled-up interventions focusing on behavior change around essential new-born care, using SHGs in India. However, the results must be considered in light of certain limitations. First, reliance on self-report outcomes on new-born health increases risk for social desirability, which may in part explain improvements in both intervention and control groups. Second, the recall bias may likely exist as the new-born care practices refer to mostly the events within first three days of baby’s birth. To reduce the recall bias, the sample for analyses in the current study was restricted to women who had 0–5 months age children. Third, the evaluation considered only the members of the group in both intervention and control geographies limiting the generalizability of the results to effects among women within groups. It may be that some of the new-born care practices are influenced by elders in women’s household and/or prevailing practices in the environment. Future research or program evaluations may consider examining the influence of other members (elders) on women in healthy practice behaviors, and the changes that have occurred among elders as a result of intervention.

## Conclusion

The present results show that simple communication messages on healthy practices through SHGs may change behaviors around new-born healthcare practices even among the women from lowest socioeconomic groups. The findings show that intervention aimed at building the knowledge of women within groups and also to mobilize women to share information with each other have several important benefits to improve new-born health. These findings indicate the utility of structured new-born healthcare messaging through SHGs in improving new-born care to further reduce the neonatal mortality, specifically in areas where one-fourth of the deliveries continue to happen at home. Further longer-term research studies are needed to assess the retention and sustainability of intervention not only for women within SHGs but also other women in the local areas, and its impact on reduction of neonatal mortality.

## Data Availability

The datasets used and/or analyzed during the current study are available from the following link: 10.7910/DVN/WPHXJK.
