# Correction: Effect of behavioral change intervention around new-born care practices among most marginalized women in self-help groups in rural India: analyses of three cross-sectional surveys between 2013 and 2016

**DOI:** 10.1038/s41372-019-0379-9

**Published:** 2019-05-02

**Authors:** Niranjan Saggurti, Akash Porwal, Yamini Atmavilas, Monika Walia, Rajshree Das, Laili Irani

**Affiliations:** 1 Population Council, New Delhi, India; 2 Bill and Melinda Gates Foundation, New Delhi, India; 3 Project Concern International, New Delhi, India

**Keywords:** Developing world, Population screening

**Correction to:** **Journal of Perinatology**

10.1038/s41372-019-0358-1

published online 02 April 2019

This article was originally published under a standard License to Publish, but has now been made available under a CC BY license. The PDF and HTML versions of the paper have been modified accordingly.

